# Giant serpentine aneurysm: Neuroradiological and neurosurgical management in a left-handed patient

**DOI:** 10.1016/j.radcr.2023.09.014

**Published:** 2023-10-04

**Authors:** Andrea Romano, Giulia Moltoni, Amedeo Piazza, Guido Trasimeni, Massimo Miscusi, Serena Palizzi, Allegra Romano, Antonino Raco, Alessandro Bozzao

**Affiliations:** NESMOS, Department of Neuroradiology, S.Andrea Hospital, University Sapienza, Rome, Italy

**Keywords:** Serpentine aneurysm, Functional MRI, Wada test, Language dominance

## Abstract

Giant serpentine aneurysms are rare huge and partially thrombosed aneurysms, with an eccentric tortuous intra-aneurysmal vascular channel. Surgical treatment is often necessary due to the great mass effect. We describe a case of a left-handed woman with a giant serpentine aneurysm of the left middle cerebral artery whose management was complex. The challenge was to exclude the aneurysm from circulation, reduce the mass effect, and, mostly, preserve the language function. Since the patient was left-handed the language dominance needed to be assessed; functional MRI (fMRI) and Wada test (WT) showed a right dominance. Surgical treatment was performed, as a complication, the patient developed left fronto-basal ischemia with a slight paresis of the right hand but without any language deficit. Our case shows the importance of a multidisciplinary team in patient management, with a pivotal role of neuroradiological functional tests in presurgical planning.

## Introduction

Giant serpentine aneurysms (GSAs) are large-size lesions (>25 mm in diameter), partially thrombosed, with an eccentric tortuous intra-aneurysmal vascular channel; their formation mechanism remains unclear, and their natural history is unknown and unpredictable [1]. GSAs are less likely to rupture due to the thick fibrous wall, however, they commonly present signs of mass effect [1–3], hence requiring treatment.

We report a case of left MCA giant serpentine aneurysm in a left-handed woman with challenging diagnosis and management.

## Case presentation

### Clinical presentation

A 45-year-old female, left-handed, with a history of cocaine abuse suffered from headaches and lipotimic events in the last 2 months. The first neurological evaluation was normal as well as a previous brain MRI acquired for headache 5 years before.

### Radiological evaluation

CT examination showed the presence of a 50 × 30 × 39 mm mixed density round mass, with a prevalent hyperdense component and slight calcifications, in the left frontal lobe, near the Sylvian scissure. The lesion was surrounded by edema, with a mass effect on the left lateral ventricle.

**Fig. 1:**
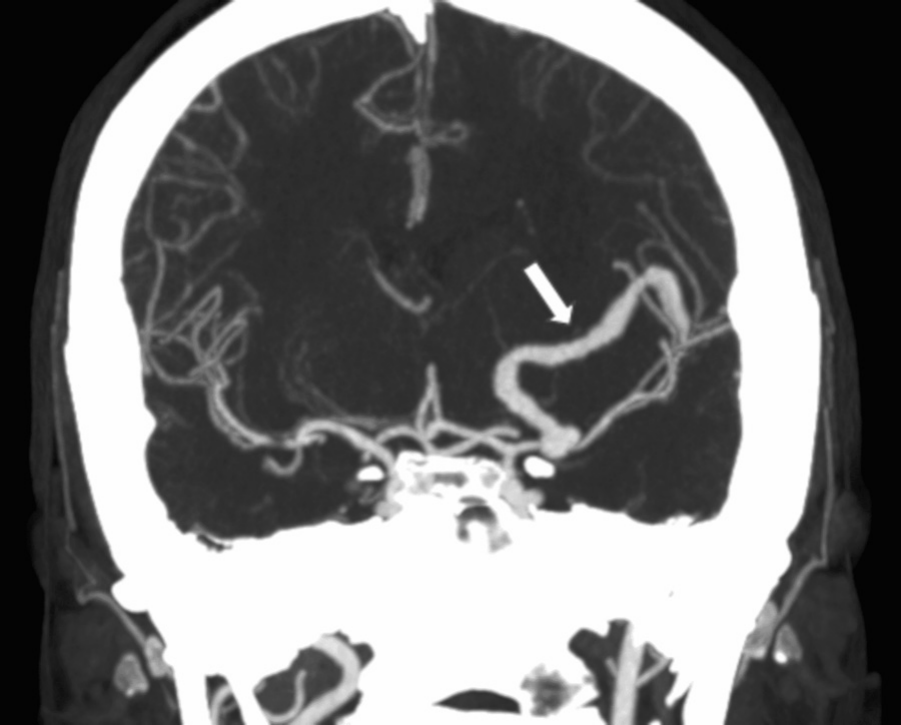
Maximum intensity projection (MIP) coronal reconstruction of a contrast-enhanced CT showing a serpentine eccentric patent vessel (arrow).

Maximum intensity projection (MIP) coronal reconstruction of contrast-enhanced CT showed a serpentine eccentric patent vessel inside the lesion (Fig. 1).

**Fig. 2:**
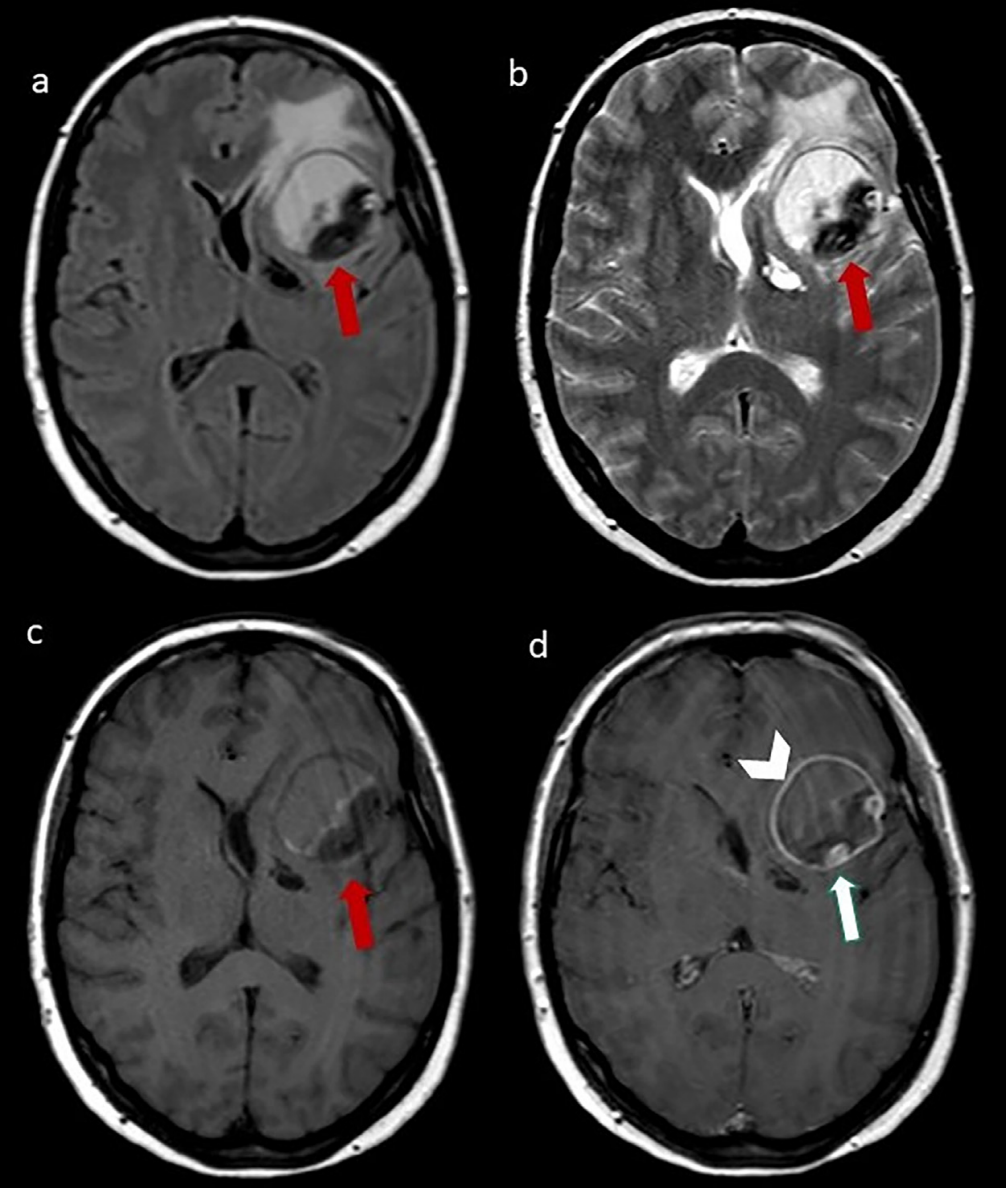
(A) Axial FLAIR, (B) axial T2- weighted image, (C) T1- weighted image, (D) T1- weighted postcontrastographic images showing a well-defined mass in the left frontal lobe characterized by inhomogeneous signal intensity on T1 and T2 weighted images, compatible with different stages of organizing hematoma, surrounded by perilesional edema. After contrast medium administration, an intralesional “serpentine” eccentric patent vessel (arrow) and a linear enhancement of the mass walls are evident (arrowhead).

**Fig. 3:**
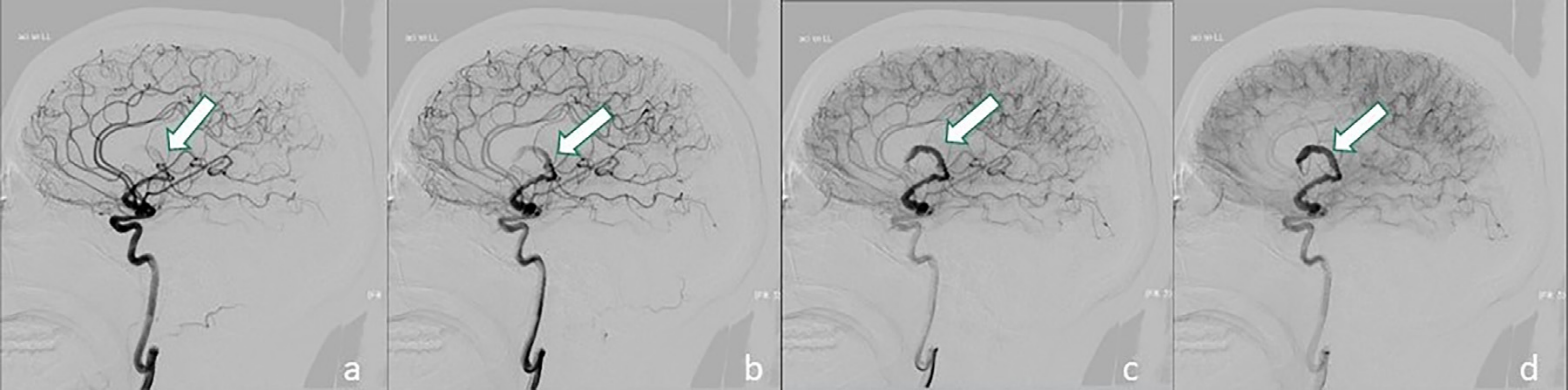
(A–D): Different phases of digital subtraction angiography (DSA) showing a partially thrombosed giant serpentine aneurysm arising from the M1 segment of the left middle cerebral artery (arrow) with distal small arterial branches reaching the fronto-basal region. These dynamic angiograms showed the slow flow inside the patent component of aneurysm with high vascular enhancement evident in the latest stage of acquisitions (D).

At MRI (1,5T, Magnetom Sonata, Siement AG, Medical Solution, Erlangen, Germany), the lesion showed well-defined margins, and inhomogeneous signal intensity on T1 and T2 weighted images, compatible with different stages of organizing hematoma and perilesional edema. After contrast-medium administration, an intralesional “serpentine” eccentric patent vessel located close to the middle cerebral artery (MCA) was evident; there was also a linear enhancement of the lesion walls (Fig. 2). Digital subtraction angiography (DSA) showed a partially thrombosed giant serpentine aneurysm arising from the M1 segment of the left MCA with distal small arterial branches reaching the fronto-basal region (Fig. 3).

**Fig. 4:**
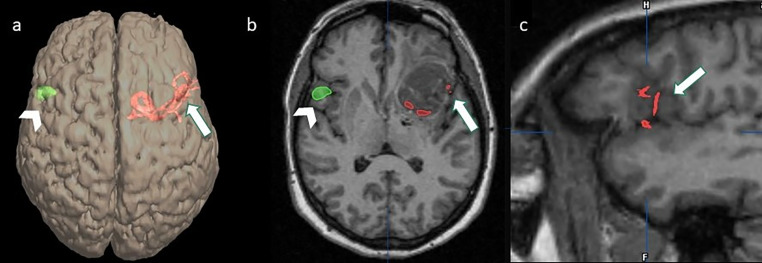
(A) Volume rendering (VR) reconstruction of volumetric T1 MPRAGE, (B) axial T1 MPRAGE, and (C) sagittal T1 MPRAGE images co-registered with DSA images and fMRI images showing vessels arising from the distal segment of the serpentine aneurysm reached the left fronto-basal cortex (A–C, arrow) and the activation of the right fronto-basal region during the task of verb generation (Broca's area) (A, B, arrowhead), suggestive of right dominance.

DSA images were co-registered with volumetric T1 MPRAGE images and bi-dimensional and volume rendering reconstructions were obtained. These reconstructions showed that vessels arising from the distal segment of the serpentine aneurysm reached the fronto-basal cortex. This leads to the hypothesis that the aneurysm arose from the superior trunk of a trifurcated MCA (Figs. 4A–C).

Because of the left-handed condition, a functional MRI examination (TE/TR = 50/3520 ms; matrix = 64×64; Field of View = 24 × 24 cm^2^; slice thickness = 3 mm; Flip Angle = 90 degrees; block design format: 8 blocks alternating task and baseline stimuli. Each block was composed of 10 images) was performed for the evaluation of hemispheric dominance. A task of verb generation led to a change in the Blood Oxygen Level-Dependent (BOLD) signal in the right fronto-basal region (Broca's area) (Figs. 4A and B) whereas a task of reading led to a BOLD signal change in the left superior temporal gyrus compatible with Wernicke area.

To confirm right dominance, a Wada Test (WT) was performed with a super-selective catheterism, an injection of 20 mg of sodium thiopental into the right and left MCA. When the right side was tested, the patient presented speech arrest, whereas this was not the case on the left side. This confirmed the right hemisphere dominance of language. An endovascular attempt to embolize the aneurysm was performed, without a successful result due to a very small aneurysm neck.

Considering the functional and angiographic results and the fact that the vascular representation distal to GSA would not allow a bypass due to its small size, surgical treatment with a proximal clipping was planned.

### Surgical treatment and postoperative course

A lumbar cerebrospinal fluid (CSF) drainage was placed preoperatively.

**Fig. 5:**
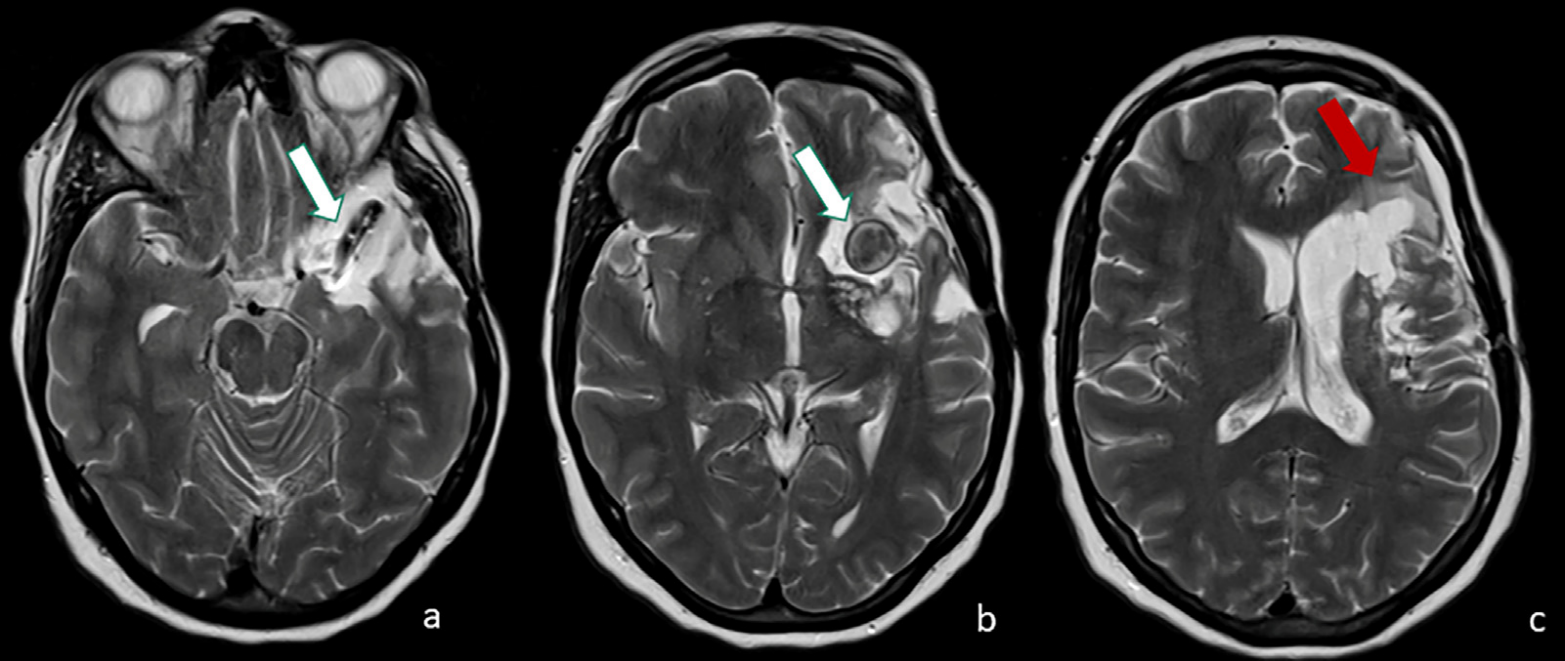
(A–C) follow-up axial T2-weighted images 10 months after the surgical treatment documented significant volumetric reduction of the residual aneurysm (A, B, white arrow) and malacic ischemic damage in the fronto-basal region (C, red arrow).

The patient was in a supine position with the head rotated 45° to the right and the left zygoma being higher than the head to favor brain retraction. A curvilinear pterional skin incision was performed. Left frontotemporal craniotomy was carried out. The small sphenoid wing was drilled. Before dural incision, 100 mg of mannitol were administrated, and 40 ccs of CSF were subtracted to prevent tightness and dissection. The Sylvian scissure was opened to expose the internal carotid artery (ICA) and the giant serpentine aneurysm of the left M1. A temporary clip-on ICA (2 minutes, 44 seconds) was placed; therefore, the aneurysm was clipped at the origin of the trunk. The wall of the aneurysm was incised, and the thrombus was aspirated with an ultrasonic suction. The patient awoke with right hemiparesis, without language deficit. MR examination showed an acute ischemic lesion of the cortical fronto-basal region with extension in deep white matter. During the third postoperative day, the patient underwent decompressive craniectomy due to worsening neurological status from increased ischemic edema in the left MCA territory. The neurological status rapidly increased and on the fifth postoperative day, a cranioplasty was performed. One month later the neurological status was normal except for slight paresis of the right hand and the patient was discharged. After 10 months, an MR documented a significant volumetric reduction of the residual aneurysm and malacic ischemic damage in the fronto-basal region (Figs. 5A–C). The mild paresis of the right hand was stable.

## Discussion

We described a rare case of GSA of left MCA in a left-handed patient with challenging diagnostic and therapeutic strategies.

A serpentine aneurysm is a giant aneurysm, extremely rare (0.6% of all aneurysms), almost completely clotted except for residual tortuous vascular channels, which are not the residual lumens of the parent artery but rather intrathrombotic channels [4].

The mechanism of GSA formation is still unclear [2] GSA may represent the expansion of a fusiform aneurysm with partial thrombosis and repeated intramural hemorrhage [2] Among several hypotheses, some authors suggested that dissection may trigger a pathological process leading to a GSA [4,5] It is known that that drug abuse, like cocaine or other neurostimulators, predisposes patients to aneurysmal formation [6] and to arterial dissection due to its effects on vascular connective tissue and its propensity to produce severe hypertension [7]. We could speculate that in our patient factors linked to cocaine abuse could contribute to the development of a dissecting fusiform aneurysm before, and GSA after. This hypothesis might be supported by the absence of vascular alterations in the MRI performed 5 years earlier.

In our patient, the therapeutic challenge was to exclude aneurysms from the circulation, reduce the mass effect, and, mostly, preserve language function.

GSAs treated conservatively may lead to neurological disorders or death. Thus, surgical treatment is mandatory but challenging [8] (either endovascular or direct clipping). In our case, surgical treatment was obliged due to the presence of a very small aneurysm neck.

From the analysis of the literature [9], only 10 out of 31 surgically treated cases of GSAs involved the left MCA but none with right cerebral dominance in a left-handed patient. In our patient, the vessel arising from the distal segment of the serpentine aneurysm reached the fronto-basal cortex through the Sylvian scissure, at the level of the Broca's region. Thus, surgical clipping had to be planned to avoid postsurgical aphasia.

We assessed hemispheric dominance by performing fMRI and selective WT. Usually, WT and electrocortical stimulation mapping (ESM) has long been considered the gold standards for assessing language dominance, but they are both invasive. Recently fMRI has emerged as the leading noninvasive modality for preoperative language mapping [10]. The verb generation task is a reliable predictor of laterality and has a high concordance with WT [10]. In our patient, fMRI showed right-sided lateralization of Broca's area. Three different conditions could justify this result. First, an atypical language dominance in the right hemisphere, indeed as reported in the literature left-handed individuals have a higher incidence of atypical language representation (right dominant or mixed) [11]; second, an underestimation of BOLD signal in the left hemisphere due to the presence of the GSA indeed in patients with vascular malformation the accuracy of fMRI remains a matter of debate [12]; third, cortical plasticity due to the presence of chronic hypoperfusion of the Broca's area related to the GSA, the so-called “mirror phenomenon” suggests that the homologous language areas in the nondominant hemisphere play similar roles when the corresponding language area in the dominant hemisphere is impaired [10]. For the above-mentioned reasons, confirmation of the right-sided lateralization of the Broca's area was obtained by means of WT. Their concordance was considered sufficient for a safe surgical clipping of GSA.

Surgical treatment is often complex due to the small aneurysm neck, difficult visualization of perforating vessels, and the rich vascular supply coursing through the surface of the aneurysm [13].

Among surgical interventions, GSA resection plus revascularization is the best approach [1].

In our case, the contralateral localization of Broca's area suggested proximal clipping of the aneurysm as the first choice. Since the distal branches arising from the GSA were considered functionally irrelevant and rather small, a bypass was not performed. This led to partial debulking of the aneurysm, with a reduction of the mass effect and the perilesional edema. However, this approach did not preserve completely motor function and the patient developed right arm hemiparesis. We can retain that this motor deficit was related to suffering MCA perforant arteries.

## Conclusions

We described a rare case of GSA arising from the left MCA in a left-handed patient where the efficacy of surgical treatment was related to a complete and double-checked neuroradiological evaluation (WT and fMRI) of Broca's cortical area. In our case, neurofunctional tests showed right-sided lateralization of Broca's area, estimating a very low risk of post-treatment aphasia. In conclusion, our case is a powerful example of the importance of a multidisciplinary team in patient management.

## Patient consent

The authors affirm that informed consent for publication of this case was obtained from the patient.
